# Development and Validation of a Dynamic Prediction Model for Massive Hemorrhage in Trauma

**DOI:** 10.1155/2022/9438159

**Published:** 2022-11-30

**Authors:** Chengyu Guo, Maolin Tian, Minghui Gong, Fei Pan, Hui Han, Chunping Li, Tanshi Li

**Affiliations:** ^1^School of Medicine, Nankai University, Tianjin 300071, China; ^2^Department of Emergency, First Medical Center, Chinese PLA General Hospital, Beijing 100089, China; ^3^School of Information Engineering, China University of Geosciences, Beijing 100083, China; ^4^School of Software, Tsinghua University, Beijing 100083, China

## Abstract

**Objectives:**

Early warning prediction of massive hemorrhages can greatly reduce mortality in trauma patients. This study aimed to develop and validate dynamic prediction models for massive hemorrhage in trauma patients.

**Methods:**

Based on vital signs (e.g., heart rate, respiratory rate, pulse pressure, and peripheral oxygen saturation) time-series data and the gated recurrent unit algorithm, we characterized a group of models to flexibly and dynamically predict the occurrence of massive hemorrhages in the subsequent *T* hours (where *T* = 1, 2, and 3). Models were evaluated in terms of accuracy, sensitivity, specificity, positive predictive value, negative predictive value, *F*1 score, and the area under the curve (AUC).

**Results:**

Results show that of the 2205 trauma patients selected for model development, a total of 265 (12.02%) had a massive hemorrhage. The AUCs of the model in the 1-h-group, 2-h-group, and 3-h-group were 0.763 (95% CI: 0.708–0.820), 0.775 (95% CI: 0.728–0.823), and 0.756 (95% CI: 0.715–0.797), respectively. Finally, the models were used in a web calculator and information system for the hospital emergency department.

**Conclusions:**

This study developed and validated a group of dynamic prediction models based on vital sign time-series data and a deep-learning algorithm to assist medical staff in the early diagnosis and dynamic prediction of a future massive hemorrhage in trauma.

## 1. Introduction 

Massive hemorrhage is one of the most serious and life-threatening complications caused by trauma and are the main cause of preventable death in trauma patients comprising 40% of trauma-related fatalities [1–4]. Compared with grossly visible massive hemorrhages, the detection of invisible massive hemorrhages is often delayed by occult bleeding. However, if the medical staff is able to identify a traumatic massive hemorrhage at an early stage and even in advance, timely and effective interventions can be performed, reducing patient disability and mortality rates and improving outcomes after severe trauma [5].

At present, most prediction methods related to traumatic hemorrhages use scoring systems based on traditional statistical methods [6–8], such as the trauma-associated severe hemorrhage (TASH) [9] and Prince of Wales Hospital/Rainer (PWH) [10] scores. Traditional scores require the manual calculation of results, which is both time-consuming and complicated. Moreover, the diversity of predictive variables makes it difficult for these scores to achieve dynamic prediction. Finally, the accuracy of these scores decreases in varying degrees as time passes and when applied to people from different regions [11].

In recent years, the development of medical noninvasive monitoring technology and equipment has achieved noninvasive, real-time, continuous, and dynamic monitoring of multidimensional vital sign information. Long-term continuous vital sign information describes the rich physiological and pathological state of the human body, and it is also an important part of clinical big data [12]. The heart rate (HR), respiratory rate (RR), blood pressure, and peripheral oxygen saturation (SpO_2_) are the basic vital signs that measure the most critical functions of the human body. A comprehensive analysis of vital signs data using time-series and machine-learning may provide substantial support in decision-making for disease prevention, diagnosis, and treatment [13–15].

This study aimed to explore the application of vital signs time-series data and deep learning, to construct a group of dynamic prediction models, and simultaneously, to predict the risk of a traumatic massive hemorrhage in the subsequent *T* hours (where *T* = 1, 2, and 3) throughout the duration of hospital care. By predicting future risk, trauma rescue teams can identify high-risk patients early, implement personalized preventive measures, initiate massive transfusion protocols, and prepare for surgical intervention in a timely manner.

## 2. Materials and Methods

### 2.1. Data Sources

The Chinese People's Liberation Army (PLA) General Hospital Emergency Trauma Database (hereafter referred to as the “trauma database”) was used in this study. The trauma database includes data on consecutive trauma patients admitted to the emergency resuscitation room from January 2015 to March 2022. The use of relevant de-identified data from the trauma database was reviewed by the Medical Ethics Committee of Chinese PLA General Hospital (ethical batch number S2021-466-01). The requirement of written informed consent was waived owing to the retrospective design of the study.

Trauma patients from the trauma database were included in this study. The exclusion criteria were as follows: (1) patients aged < 16 years; (2) patients with second or further repeat admissions after trauma (to rule out massive hemorrhage caused by nontraumatic factors); (3) patients with a data loss rate of more than 20% (to reduce the deviation in the process of data filling); and (4) patients whose time-series length of vital signs data in the data extraction interval were less than three records (to ensure that there were enough time-series data for model training).

### 2.2. Outcomes

In this study, a massive hemorrhage represented the outcome variable; however, there is no authoritative standard for the screening criteria for a massive hemorrhage. By consulting previous literature [16, 17] and combining our findings with clinical practice, we determined the screening criteria as: (1) a massive transfusion of three or more units of red blood cells within one hour at any time during the first 24 hours after admission, (2) embolization or hemostatic surgery within 24 hours after admission. Patients were classified in the “massive hemorrhage group” if they met either of the aforementioned conditions; otherwise, they were classified in the “nonmassive hemorrhage group”.

### 2.3. Predictive Variables

This study used the HR, RR, pulse pressure (PP), and SpO_2_ as predictive variables. Models were developed based on the time-series data of the four vital signs. Generally, data on vital signs from the trauma database was based on electrocardiogram monitor and pulse oximeter measurements, which were recorded every hour throughout in-patient care. For patients in the massive hemorrhage group, “study section time” was defined as the time point at which the screening criteria was first met. For patients in the nonmassive hemorrhage group, the recording time of the last vital sign data before the time point of 24 h after admission was considered the study section time. Three time intervals (1–13 h, 2–14 h, and 3–15 h) before the study section time were regarded as the three “data extraction intervals”. After the vital signs time-series data in these three intervals were extracted, three models were developed to predict the risk of a massive hemorrhage in the future, at one, two, and three hours later, respectively (hereinafter, referred to as “1 h group”, “2 h group”, and “3 h group”) (Supplementary Figure 1); for instance, if a trauma patient was transfused 4 units of red blood cells within the 15th hour past admission, the 15th hour after admission was considered as the study section time, and the 2–14 h, 1–13 h, and 0–12 h time periods after admission constituted the three data extraction intervals.

### 2.4. Model Development and Validation

We used a gated recurrent unit (GRU) deep-learning algorithm to develop and validate the prediction models [18]. The use of the GRU is a variant of recurrent neural networks which was proposed by Cho et al. in 2014. It has a simple network structure and solves the problem of gradient disappearance that occurs with traditional recurrent neural networks. GRU has an excellent performance in sequential data processing, classification, and prediction. In model training, the early-stop mechanism was used to avoid overfitting. In case the current minimum verification error was not updated in 10 consecutive epochs, the training was stopped, and the maximum epoch was 200. The deep-learning model used the cross-entropy loss function. The parameters of the model are indicated in Supplementary Table 1. A ten-fold cross-validation was used for model validation. The accuracy, sensitivity, specificity, positive predictive values, negative predictive values, *F*1 scores, and areas under the curve (AUC) were used to evaluate the models' performance.

### 2.5. Statistical Analyses

Python 3.8.5 (Python Software Foundation) was used to develop and validate the models. The GPU model used was the GeForce RTX 2080 Ti (American NVIDIA Corporation), with an 11 GB video memory capacity. Stata 17 was used for the statistical analysis. The quantitative data were expressed by means (SDs), and a *t*-test or one-way ANOVA was performed for differential analysis. The categorical data were expressed using *n* values (%) and compared using the chi-square tests. The level of statistical significance was set at *P* < 0.05.

## 3. Results

### 3.1. Comparison of Baseline Characteristics

**Figure 1:**
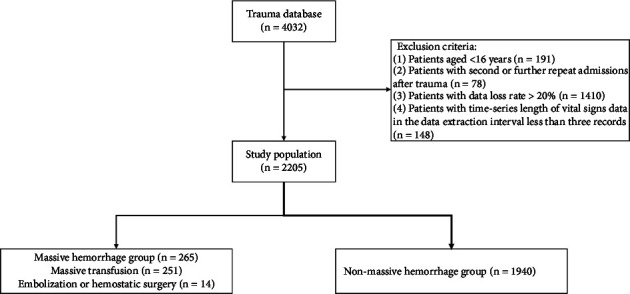
Patient selection flow chart.

A total of 4032 patients with trauma were included in the trauma database. After screening according to the exclusion criteria, 2205 patients were included in the study, with an average age of 47.42 years (SD, 17.40), with males accounting for 77.60% of the patients included. Among all patients included, 265 (12.02%) met the outcome variable of a massive hemorrhage (Figure 1).

Data shows the patients in the massive hemorrhage group were more likely to have an increased HR and a decreased PP and SpO_2_; there were no significant differences in sex and age between the massive hemorrhage group and the nonmassive hemorrhage group (Table 1).

### 3.2. Development and Validation of Dynamic Prediction Models for Massive Hemorrhages in Trauma Settings

**Figure 2:**
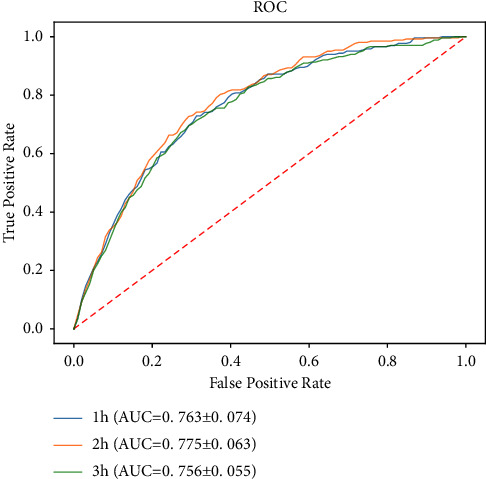
Comparison of the ROC curves of the GRU models in 1 h, 2 h, and 3 h groups. ROC: receiver operating characteristic curve; AUC: area under the curve; GRU: gated recurrent unit.

Table 2 shows the seven evaluation indexes of the models, with AUCs of 0.763 (95% CI: 0.708–0.820), 0.775 (95% CI: 0.728–0.823), and 0.756 (95% CI: 0.715–0.797) in the 1 h, 2 h, and 3 h groups, respectively. The receiver operating characteristic (ROC) curves and AUC differences of the models in the 1 h, 2 h, and 3 h groups were compared [Figure 2]. There were no significant differences in the AUCs among the three groups (*P*=0.572).

**Figure 3:**
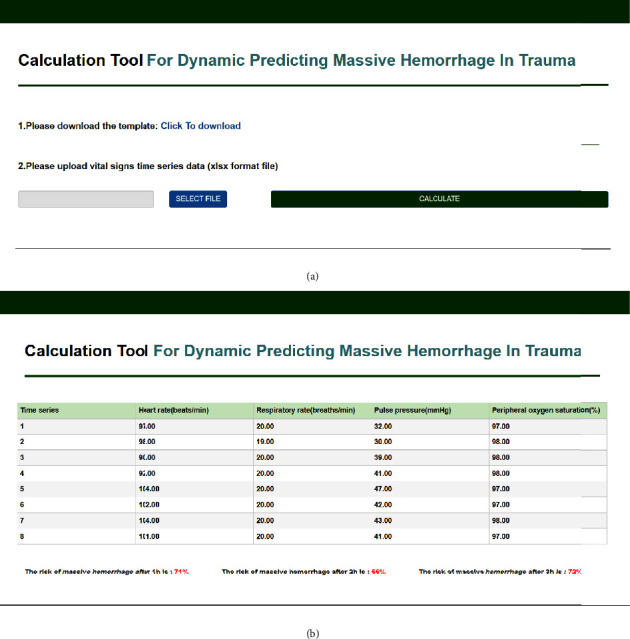
The web calculator for the dynamic prediction models for a future massive hemorrhage in trauma. (a) Data entry page; (b) forecast results page. Click the ‘Click to download' link in Figure 3(a) to download the excel template, import the vital signs time-series data according to the template format, click the ‘SELECT FILE' button to select the file to be uploaded, and finally, click the “CALCULATE” button for risk prediction. The imported vital signs time-series data are shown at the top of Figure 3(b), and the prediction probability of massive hemorrhage in trauma in the future 1, 2, and 3 hours is shown below, in the range of [0, 1]. The higher the value, the greater the risk of massive hemorrhage.

To facilitate use by public and medical personnel and further validate our model, we developed a web calculator (http://82.156.217.249:5008/) including a data entry page [Figure 3(a)] and the forecast results page [Figure 3(b)], which supported the early assisted diagnosis and dynamic prediction of a future massive hemorrhage in patients with trauma. To promote the practical application of the model, we further used the models in the emergency department information system, which supported the automatic extraction of the vital signs time-series data, as well as the efficient and intelligent prediction of a traumatic hemorrhage.

## 4. Discussion

Based on the vital signs time-series data in the trauma database, we developed a group of deep-learning models to enable the dynamic prediction of the risk of a massive hemorrhage at the next three time points. Moreover, we designed an open and accessible data interface to validate our model and for use by medical staff and the public. Finally, we used the models within the hospital information system to help clinicians in early identification, dynamic prediction, and decision-making for patients with a massive hemorrhage.

In this study, the time-series data of the vital signs were used to develop models. The models had several advantages. First, as the vital sign data can be obtained easily and quickly in both prehospital and in-hospital environments, medical staff can record this data regularly and with ease and input the data into the model for risk prediction without waiting for laboratory test results such as routine blood tests, coagulation function tests, or results from imaging studies such as ultrasound and computed tomography, which are all helpful factors in improving the timeliness of the model [19]. Second, the vital sign indices can be monitored noninvasively and repeatedly, ensuring that the model can be recalculated continuously, during prehospital first-aid interventions or emergency triage, providing valuable information on patients' responses to treatment, and thus allowing medical professionals to modify treatment plans more efficiently [13]. Finally, the three models predicted the risk of traumatic massive hemorrhage simultaneously in the next one, two, and three hours, achieving a dynamic prediction and providing extensive information regarding injury changes [14].

Presently, the prediction models related to traumatic massive hemorrhages that have been reported in the literature have mainly used massive transfusions as the outcome variable [6–8]. However, a massive transfusion cannot explain all the clinically important results related to a massive hemorrhage. For example, before the standard for a massive transfusion is reached, patients with a massive hemorrhage may have received a hemostatic intervention. Therefore, a competing risk bias may occur when a massive transfusion is used alone as an outcome variable in a prediction model [16]. In addition, the traditional definition of a massive transfusion of ≥10 units of red blood cells within 24 hours should be considered obsolete. The critical administration threshold that defines the receipt of three or more units of red blood cells during a single hour anytime during the first 24 hours of arrival can identify patients with a massive hemorrhage more accurately and minimize survivor bias [20]. Therefore, our study used the definition of a massive transfusion or “embolization or hemostatic surgery” within 24 hours of admission as the screening condition for a massive hemorrhage in trauma, emphasizing the severity of the hemorrhage rather than the amount of blood transfused, and using the modern definition of a critical administration threshold instead of the traditional definition of a massive transfusion.

To help the public and medical staff use the models more conveniently, we developed a web calculator that provides a user-friendly interface. After inputting the predictive variables, the risk of a massive hemorrhage in patients with trauma at the next three time points can be predicted dynamically. These results will help clinical decision-makers understand the patient's condition and prepare appropriate treatment strategies. Although the web calculator can meet the access needs of the public in different countries and regions, for emergency department clinical staff that have a heavy workload and operate at a fast pace, inputting the time-series data of the four vital signs manually remains time-consuming and complex; more so when the time-series is long. It is therefore easy for human errors to occur. This limits the practical application of the models to a great extent. To reduce the time and labor cost of using the models, we used them within the hospital emergency department information system. As part of the computerized clinical decision-support system, it enabled the automatic, continuous, efficient, and dynamic prediction of a traumatic hemorrhage, which can assist clinicians in early diagnosis and dynamic monitoring, may improve diagnosis and treatment practices of residents and attending physicians, and thus, improve medical services.

This study had several limitations. First, the study used two screening strategies, including massive transfusion and embolization or hemostatic surgery to cover as many trauma patients with massive hemorrhage as possible. A small number of patients were not included in the study population because they either refused to undergo the examination and treatment or died before receiving treatment. We will include trauma patients with massive hemorrhage-related deaths in future studies to further reduce survivor bias. Second, the prediction models for a massive hemorrhage in trauma can only guide the doctor's clinical decision-making process and cannot replace the doctor's clinical judgment and the results of other diagnostic tests. Finally, as this was a retrospective observational study, prospective validation is still needed. In future studies, it will be necessary to determine whether the use of dynamic prediction models for a traumatic hemorrhage can reduce waiting times before the implementation of massive transfusion protocols or surgical intervention. Furthermore, it will also be important to determine its impact on the prognosis of patients with trauma.

## 5. Conclusions

In this study, a group of dynamic prediction models were developed and validated based on the vital signs time-series data and the GRU deep-learning algorithm to assist in the early diagnosis and dynamic prediction of a future massive hemorrhage in trauma. The models were used in the web calculator and the hospital information system, which promoted their clinical application.

## Figures and Tables

**Table 1:** Comparison of baseline characteristics.

Characteristics	Totals	Massive hemorrhage		*P* values
		Yes	No	
No.	2205	265	1940	
Male, n (%)	1711 (77.60%)	196 (74.0%)	1515 (78.1%)	0.130
Age, mean (SD), y	47.42 (17.40)	47.60 (16.10)	47.39 (17.57)	0.850

Vital signs, mean (SD)				
Heart rate, beats/min	93.10 (21.64)	104.35 (23.80)	91.96 (21.08)	<0.001*∗*
Respiratory rate, breaths/min	19.58 (2.20)	19.58 (2.40)	19.58 (2.17)	0.890
Pulse pressure, mmHg	49.08 (14.02)	46.34 (13.54)	49.36 (14.04)	<0.001*∗*
Peripheral oxygen saturation, %	97.50 (2.85)	97.00 (3.95)	97.56 (2.70)	<0.001*∗*

The *P* values pertain to the result of differential analysis between the massive hemorrhage and nonmassive hemorrhage groups. Quantitative data were expressed by means (SD) and the differences were analyzed using the *t*-tests. Classified data were expressed using *n* values (%) and the differences were analyzed using a chi-square test. *∗*indicates that the difference is statistically significant. SD: standard deviation.

**Table 2:** Comparison of the models' effects in 1 h, 2 h, and 3 h groups.

	ACC	SEN	SPE	PPV	NPV	F1	AUC
1 h group	0.683 ± 0.029	0.729 ± 0.128	0.677 ± 0.036	0.235 ± 0.030	0.949 ± 0.024	0.355 ± 0.049	0.763 ± 0.074
2 h group	0.687 ± 0.034	0.750 ± 0.161	0.678 ± 0.050	0.240 ± 0.037	0.954 ± 0.026	0.362 ± 0.059	0.775 ± 0.063
3 h group	0.685 ± 0.032	0.714 ± 0.135	0.681 ± 0.033	0.234 ± 0.044	0.946 ± 0.025	0.352 ± 0.065	0.756 ± 0.055

ACC: accuracy; SEN: sensitivity; SPE: specificity; PPV: positive predictive value; NPV: negative predictive value; F1: F1 score; AUC: area under the curve.

## Data Availability

The data are available from the Chinese PLA General Hospital Emergency Trauma Database, although restrictions apply to the availability of these data, which were used under license for the current study, and so are not publicly available. Data are however available from the corresponding authors upon reasonable request and with permission of the Chinese PLA General Hospital.

## Supplementary Materials

Supplementary Materials
*Supplementary Table 1.* Parameter settings of the GRU model. *Supplementary Figure 1*. Schematic diagram of the vital signs time-series data extraction. (a) 1 h group; (b) 2 h group; (c) 3 h group.
