# Supplementary material for: Development and Validation of a Dynamic Prediction Model for Massive Hemorrhage in Trauma
Source: Emerg Med Int. 2022 Nov 30;2022:9438159. doi: 10.1155/2022/9438159 (PMC9729037; doi:10.1155/2022/9438159)
Supplement: Supplementary Materials — Supplementary Table 1. Parameter settings of the GRU model. Supplementary Figure 1. Schematic diagram of the vital signs time-series data extraction. (a) 1 h group; (b) 2 h group; (c) 3 h group. [file 9438159.f1.zip › Supplementary Table 1 (1).docx]

Supplementary Table 1. Parameter settings of the GRU model.

| Parameter | Setting |
| --- | --- |
| hidden_size | 32 |
| layer_num | 1 |
| learning rate | 0.0003 |
| batch size | 128 |

GRU: gated recurrent unit.
