# Supplementary figures and images for: Development and Validation of a Dynamic Prediction Model for Massive Hemorrhage in Trauma
Source: Emerg Med Int. 2022 Nov 30;2022:9438159. doi: 10.1155/2022/9438159 (PMC9729037; doi:10.1155/2022/9438159)

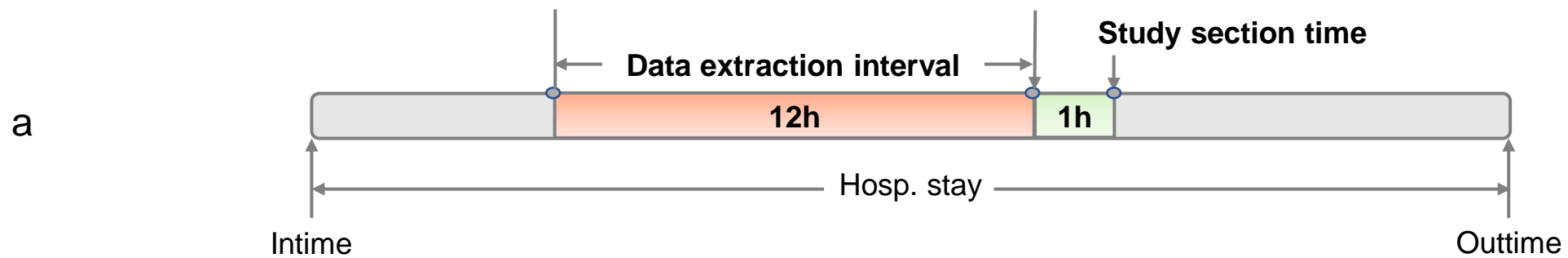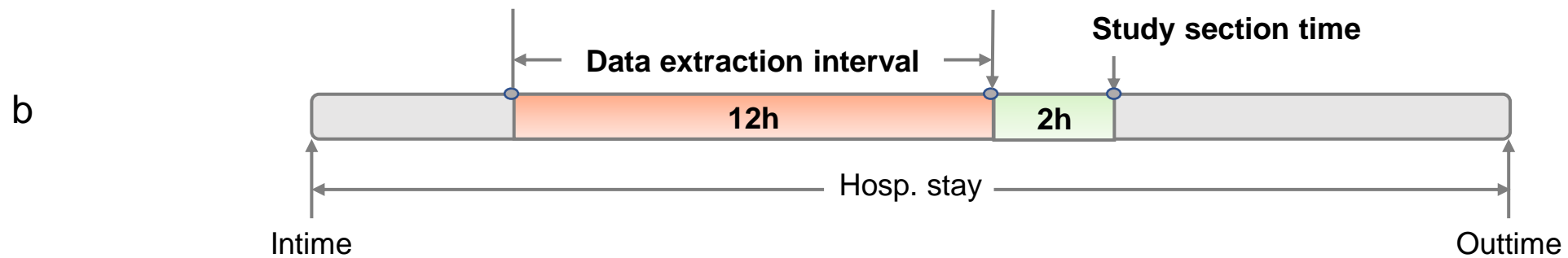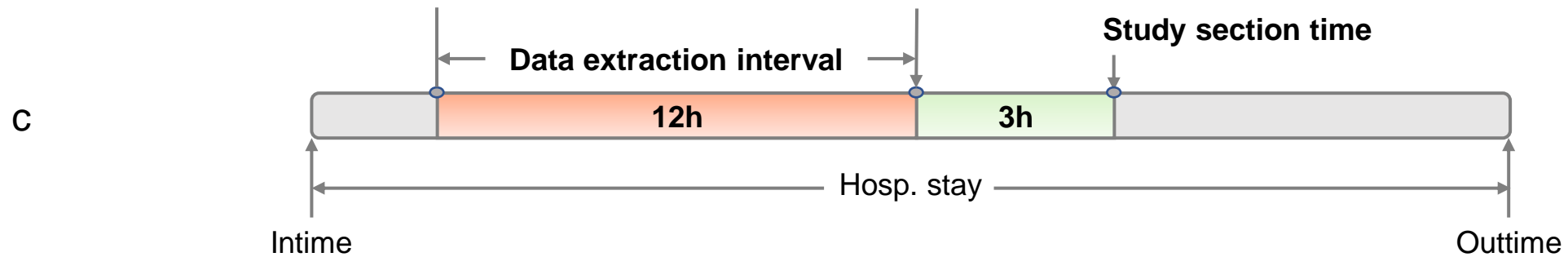

Supplement: Supplementary Materials — Supplementary Table 1. Parameter settings of the GRU model. Supplementary Figure 1. Schematic diagram of the vital signs time-series data extraction. (a) 1 h group; (b) 2 h group; (c) 3 h group. [file 9438159.f1.zip › Supplementary Figure 1 (1).pdf]
